# Temporal changes in respiratory adenovirus serotypes circulating in the greater Toronto area, Ontario, during December 2008 to April 2010

**DOI:** 10.1186/1743-422X-10-15

**Published:** 2013-01-07

**Authors:** Kaniza Zahra Abbas, Ernesto Lombos, Venkata R Duvvuri, Romy Olsha, Rachel R Higgins, Jonathan B Gubbay

**Affiliations:** 1 Public Health Ontario Labs, 81 Resources Rd, M9P3T1, Toronto, ON, Canada; 2 University of Toronto, 105 George St, M5A2N4, Toronto, ON, Canada; 3 Mount Sinai Hospital, 600 University Avenue, M5G1X5, Toronto, ON, Canada; 4 The Hospital for Sick Children, 555 University Avenue, M5G1X8, Toronto, ON, Canada; 5 Centre for Disease Modelling, York University, M3J1P3, Toronto, ON, Canada

**Keywords:** Human adenoviruses, Serotyping, Molecular typing, Phylogeny

## Abstract

**Background:**

Certain adenovirus serotypes cause severe infections, especially in children. It is important to monitor temporal changes in serotypes causing clinical disease. The objective of this study was to document circulating respiratory adenovirus serotypes by sequencing adenovirus culture isolates from the Greater Toronto Area, Ontario, during December 2008 to April 2010.

**Methods:**

Nucleic acid extraction was performed on 90 respiratory tract adenovirus culture isolates. PCR amplification was conducted with primers targeting the adenovirus hexon gene hypervariable region 7. Sanger sequencing and phylogenetic analyses were performed to determine serotype identities.

**Results:**

Among 90 clinical respiratory isolates sequenced, eight different serotypes were identified. Serotype 3 (34, 38%), serotype 2 (30, 30%), and serotype 1 (14, 16%) isolates were most common; serotypes 5, 6, 11, 17 and 21 were also observed. Seventeen (50%) of the 34 HAdV-3 isolates were identified between December 2008 and February 2009, while none were identified from December 2009 to February 2010. Between December 2008 and April 2009, the two most common serotypes were HAdV-3 and HAdV-2, detected in 18 (53%) and 8 (24%) of the 34 cultures isolates, respectively. However, from December 2009 to April 2010, there was an increase in HAdV-2, which became the most prevalent serotype, detected in 10 (50%) of the 20 isolates identified (p = 0.05).

**Conclusions:**

There was a gradual shift in prevailing adenovirus serotypes during the 17 month study period, from predominantly HAdV-3 to HAdV-2. If an adenovirus vaccine were to be broadly implemented, multiple serotypes should be included.

## Background

Human adenovirus (HAdV) belong to the *Adenoviridae* family and the *Mastadenovirus* genus [1]. They are structurally icosahedral, non-enveloped, double-stranded, linear DNA viruses [1]. HAdV are responsible for causing a variety of diseases including upper and lower respiratory infections, conjunctivitis, pneumonia, gastroenteritis and hemorrhagic cystitis [2]. More severe disease is observed among children, the elderly and immunocompromised persons [2,3]. Transmission of HAdV can occur by direct contact, small droplet aerosols, through the water system, and via the fecal-oral route [1,4].

Currently there are 52 known HAdV serotypes, each classified into one of the seven different species ranging from A to G, based on immunological and biochemical characteristics [1]. Respiratory tract infections are caused by serotypes in species B, C and E [1]. There are 10 HAdV serotypes that have been associated with pneumonia and febrile respiratory illness (FRI) [5]. However, only HAdV-3, HAdV-4, HAdV-7 and HAdV-21 have been found to cause outbreaks of both FRI and acute respiratory disease (ARD) [5,6]. Determining prevailing serotypes in a community assists in better understanding the epidemiology of disease caused by individual serotypes in order to control its spread.

A recent study undertaken at Public Health Ontario Laboratories (PHOL) described adenovirus serotypes circulating in the Greater Toronto Area (GTA), Ontario from September 2007 to June 2008. The predominant serotypes observed were HAdV-3 (46%), HAdV-2 (26%), HAdV-1 (18%) and HAdV-21 (5%); HAdV-14 was not documented [3]. The current study was conducted to document whether circulating adenovirus serotypes change temporally within the same geographic area, and to what degree these changes have occurred between December 2008 and April 2010 in the GTA of Ontario.

## Methods

### Sample source and study isolates

PHOL performs a large proportion of primary respiratory viral testing for the province of Ontario from a variety of clinical settings including ambulatory, hospital and outbreaks. Following the testing procedure for respiratory specimens, nasopharyngeal swabs (NPS) from ambulatory and hospitalized, non-intensive care unit (non-ICU) patients received by PHOL-Toronto are cultured for virus isolation in two cell lines. One line is monkey kidney cells [either Rhesus (RMK) or African green monkey kidney cells (AGMK)], and the other is WI-38 human embryonic lung fibroblast cells (Diagnostic Hybrids, Inc. Athens, Ohio, USA). Cell lines showing cytopathic effect are stained with a blend of murine monoclonal antibodies (MAbs) directed against seven respiratory viruses plus separate DFA reagents, each consisting of MAb blends directed against a single respiratory virus, including adenovirus (D3 Ultra™ DFA Respiratory Virus Screening & ID Kit, Diagnostics Hybrids). Samples submitted from patients in outbreak or ICU settings undergo multiplex molecular testing for respiratory viruses, and only a proportion also have viral culture done.

All available adenovirus culture isolates, identified from December 2008 to April 2010 at PHOL, were selected for this study. Samples that underwent molecular testing, but not viral culture, were not included. The period from December until April was regarded as winter/early spring. Isolates were derived from persons ranging in age from newborns to adults (0–88 years), with 73 (81%) from persons under 18 years of age, and 17 (19%) from persons 18 years of age or older.

This study was considered exempt from University of Toronto’s Health Sciences Research Ethics Board review as it involved deidentified respiratory tract samples that were tested as part of a clinical virology service provided by PHOL. All test-positive samples and a proportion of test negative samples are stored for possible further laboratory-based surveillance work. Samples and isolates included in this study were analyzed as part of the routine respiratory viral molecular surveillance program that supports Ontario’s Ministry of Health and Long-Term Care.

### Molecular serotyping

The methods closely follow those developed by Yeung *et al*. [3]. From each tissue culture isolate, a 250 μL aliquot underwent total nucleic acid extraction using the bioMérieux NucliSENS® easyMAG® protocol (bioMérieux Inc. Marcy I’Etoile, Rhone, France); a total elution volume of 55 μL per isolate was obtained.

The primers used for the polymerase chain reaction (PCR) were taken from Sarantis *et al.*, targeting the highly conserved regions flanking the hypervariable region-7 (HVR-7) of the HAdV hexon gene [2]. These primers generated amplicons ranging from 605 to 629 base pairs from known adenovirus serotypes [2]. For each reaction there was a total volume of 45 μL, composed of 5 μL of forward primer, 5 μL of reverse primer, 20 μL of the Qiagen® Quantitect Multiplex Master Mix (Qiagen, Inc. Venlo, Limburg, Netherlands), 10 μL of RNase free water and 5 μL of DNA template. PCR was performed on the Bio-Rad iCycler iQ™ Real-Time PCR Detection System (Bio-Rad Laboratories, Inc. Hercules, CA, USA). PCR reaction started with 3 min of initial denaturation step at 94°C, and proceeded with 34 cycles of denaturation for 1 min at 94°C, annealing for 1 min at 60°C and elongation for 1 min at 72°C. This was followed by a final 10 min incubation step at 72°C. Five microliters of the PCR product was then mixed with 1 μL of 6X Fermentas® loading dye and loaded on to a 1% agarose gel with ethidium bromide along with a Fermentas® GeneRuler™ 100 base pair (bp) DNA ladder (Thermo Fisher Scientific, Waltham, MA, USA). The gel was run for 30 min at 120 volts using a Bio-Rad PowerPac Basic™ Power Supply, and Bio-Rad Molecular Imager® Gel Doc™ XR+ Imaging System was used to visualize the band of nucleic acid at the 600 bp mark.

BigDye™ Terminator Sequencing Kit (Applied Biosystems, Inc. Foster City, CA, USA) was used to label the DNA. Two reactions per sample were conducted, one with the forward primer and the second with the reverse primer. Each reaction had a total volume of 20 μL, with 2 μL of BigDye™ terminator ready mix, 3 μL of 5x Sequencing Buffer, 2 μL of forward/reverse primers, 8 μL of RNase free water and 5 μL of PCR product. The amplification parameters for the BigDye™ PCR were as follows: the initial denaturation step was 1 min at 96°C, followed by 25 cycles of 10 second denaturation at 96°C, 5 seconds of annealing at 55°C and 4 min of elongation at 60°C.

The excess BigDye™ Terminator components were cleaned using the Qiagen® DyeEx 2.0 Spin Kit protocol. The liquid end product was dried and re-suspended with Applied Biosystems Hi-Di™ formamide and loaded onto the ABI® Prism™ 3100 Genetic Analyzer. Sequences obtained were analyzed using FinchTV™ (Geospiza, Inc. Seattle, WA, USA), and each serotype was designated using NCBI BLAST scores. Isolates with a match in GenBank with 98% or greater Max Identity score were categorized as belonging to the same serotype [2].

Raw data was entered into an Excel 2010 spreadsheet (Microsoft Co. Redmond, WA, USA) and subsequently analyzed using IBM® SPSS® Statistics 20 (International Business Machines Co. Armonk, NY, USA). Proportions were compared by χ^2^ test; all hypothesis tests were two-tailed, and p <0.05 was considered significant.

### Phylogenetic analysis

HAdV species specific phylogenetic trees were constructed using the maximum likelihood method. Statistical significance of the tree topology was tested by bootstrapping (1,000 replicates) using MEGA 5.05 software [7]. Evolutionary distances were derived using the Kimura-2 parameter method [8]. GTA HAdV nucleotide sequences and the corresponding reference sequences were included in the phylogenetic analysis after multiple alignments using the ClustalW algorithm.

### Nucleotide sequence accession numbers

The HAdV sequences that are reported here were all deposited onto the GenBank sequence database with accession numbers JX901190 to JX901279.

## Results

### HAdV serotype identity and distribution

Between December 2008 and April 2010, HAdV was detected in 229 (0.9%) of the 24,406 respiratory samples processed at PHOL-Toronto by culture and/or molecular testing; 90 (44%) of the 207 documented culture isolates were still in storage and of sufficient volume (250 μL) to be sequenced. Table 1 shows the distribution of serotypes identified during the course of this study. Our findings indicate that the most prevalent serotypes were HAdV-3 and HAdV-2, followed by HAdV-1. We also detected serotypes HAdV-5, HAdV-6, HAdV-11, HAdV-17 and HAdV-21. Males and females were equally represented. Significantly more (56, 62%) isolates were from children ≤ 4 years of age than from persons > 4 years of age (34, 38%; p <0.001). HAdV-1 was only observed in children ≤ 4 years of age, and HAdV-2 was also significantly more common in this age group (p <0.001; see Table 1). Among the 17 isolates detected throughout the study period from persons aged ≥ 18 years, the serotypes detected include HAdV-3 and HAdV-21 (6, 35% each), HAdV-2 and HAdV-11 (2, 12% each), and HAdV-17 (1, 6%). HAdV-11, HAdV-17 and HAdV-21 were not detected in children [(age < 18 years); see Table 1].

**Table 1:** Serotype identities and sex and age distribution of source patients of 90 human adenovirus respiratory culture isolates identified in the Greater Toronto Area, Ontario, from December 2008 to April 2010

**Species**	**Serotype**	**No. of Isolates**	**Sex**		**Age**		
			**M**	**F**	**≤ 4**	**4 < to < 18**	**≥ 18**
B	3	**34 (38%)**	18	16	17 (50%)	11 (32%)	6 (18%)
	11	2 (2%)	2	0	0	0	2
	21	6 (7%)	5	1	0	0	6 (100%)
C	1	14 (16%)	5	9	14 (100%)	0	0
	2	**30 (33%)**	14	16	22 (73%)	6 (20%)	2 (7%)
	5	2 (2%)	0	2	2	0	0
	6	1 (1%)	1	0	1	0	0
D	17	1 (1%)	0	1	0	0	1
Total			45 (50%)	45 (50%)	56 (62%)	17 (19%)	17 (19%)

### Geographical distribution

**Figure 1:**
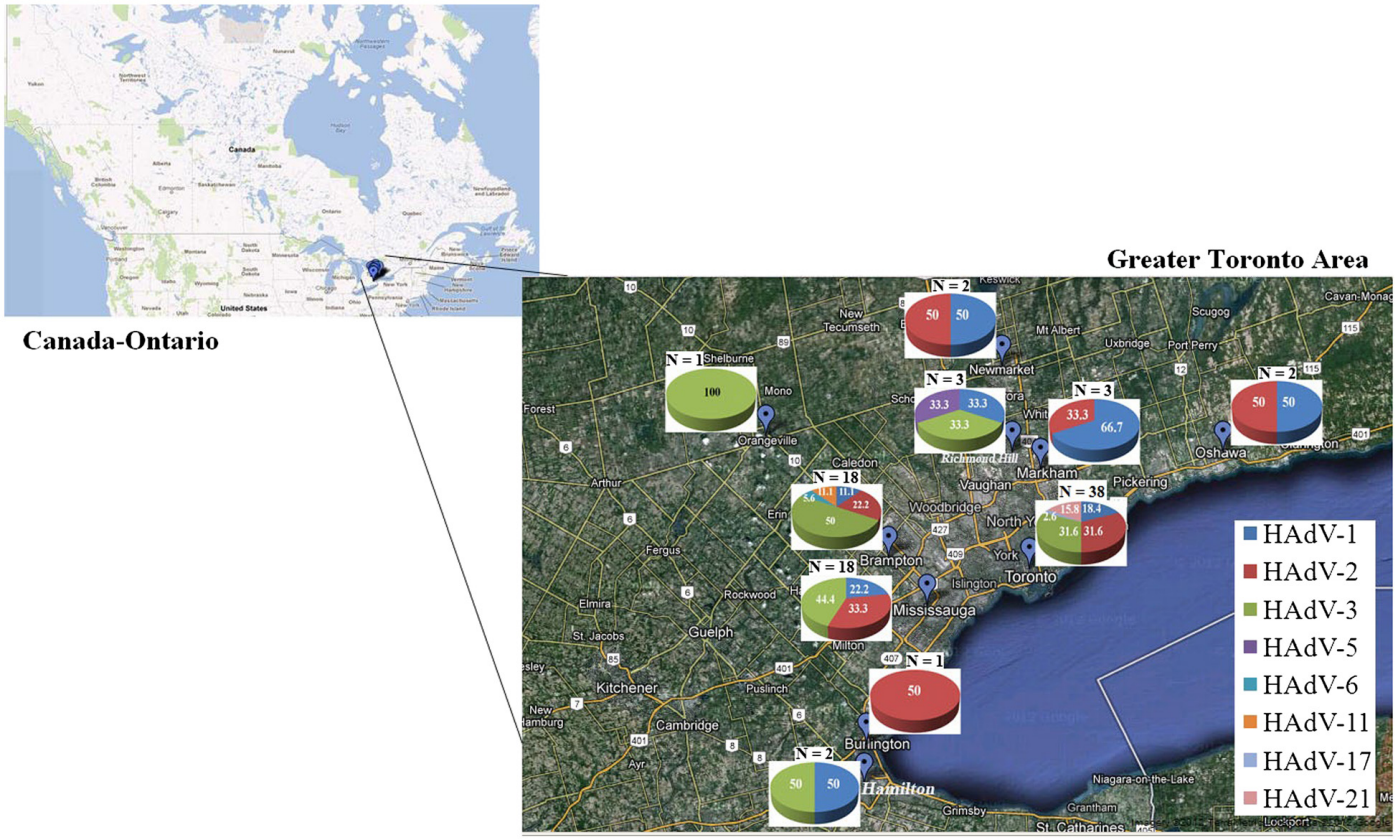
**Geographical distribution of human adenovirus serotypes causing respiratory infection in the Greater Toronto Area, Ontario, from December 2008 to April 2010.** Pie charts represent the HAdV serotypes identified in each region, and their subsequent serotypic identities. The numbers within the pie charts are percentages from the total of each serotype. The “N” number above each chart represents the total number of samples that were found in that area.

Figure 1 illustrates the geographic distribution of the different HAdV serotypes across the GTA. The majority of the HAdV cases that occurred from December 2008 to April 2010 were in the cities of Toronto (38, 42%), Brampton and Mississauga (18, 20% each). They were also the three most common cities of origin among all 229 HAdV positive samples tested at PHOL. Toronto (109, 48%) and Mississauga (48, 21%) contributed similar proportions of cases, but Brampton (27, 12%) had less when compared to the subset of 90 sequenced isolates. Also, Figure 1 shows that HAdV-3 and HAdV-2 were widely distributed throughout the GTA, while the three serotypes that were detected only in adults, HAdV-21, HAdV-17 and HAdV-11, were each restricted to a single city. HAdV-21 and HAdV-17 were only identified in Toronto, and HAdV-11 was only found in Brampton.

### Temporal distribution

**Figure 2:**
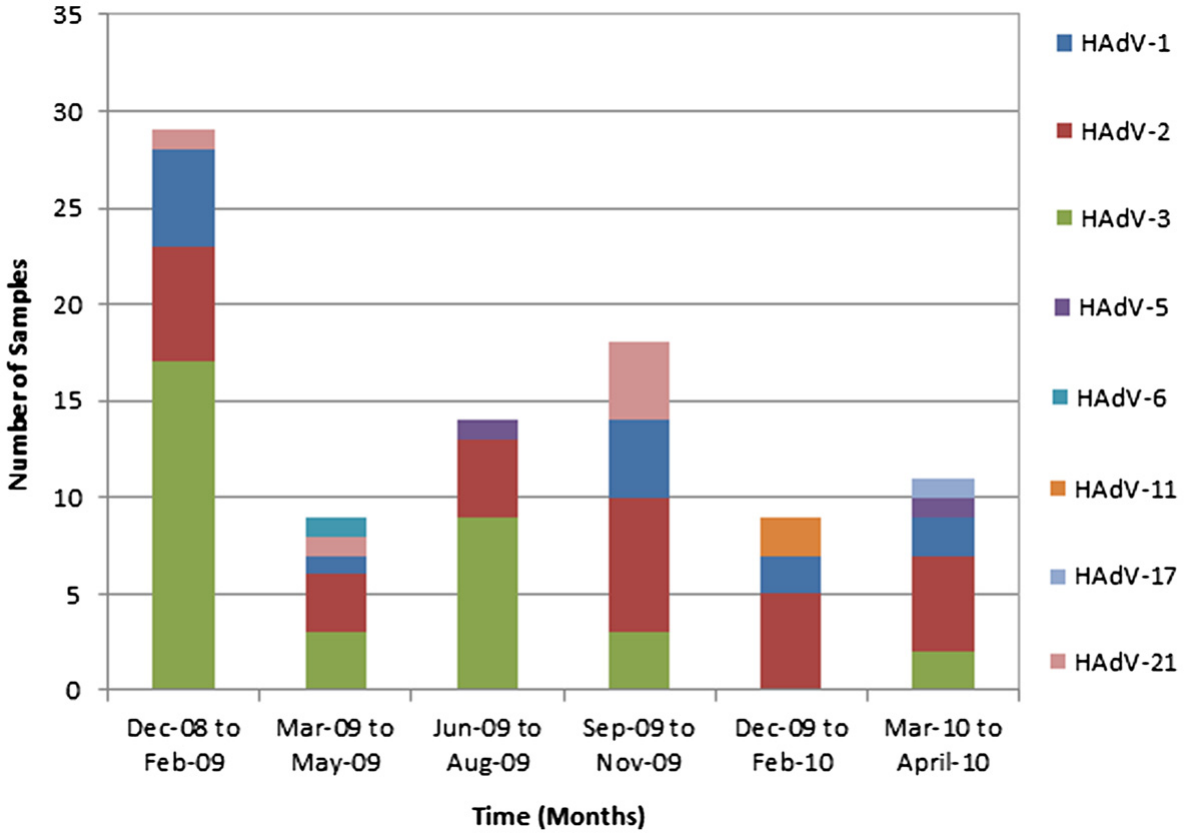
Temporal distribution of human adenovirus serotypes causing respiratory infection in the Greater Toronto Area, Ontario, from December 2008 to April 2010.

Figure 2 demonstrates the temporal changes in HAdV serotypes over the course of the 17 month study period. Between December 2008 and April 2009, the two most common serotypes were HAdV-3 and HAdV-2, detected in 18 (53%) and 8 (24%) of the 34 cultures isolates, respectively. However, from December 2009 to April 2010, there was an increase in HAdV-2, which became the most prevalent serotype, detected in 10 (50%) of the 20 isolates identified (p =0.05). Also, of the total 34 HAdV-3 isolates identified, 17 (50%) were found between December 2008 and February 2009, while none were detected between December 2009 and February 2010 (p <0.001). In addition, during December 2008 to February 2009, 6 (21%) of the 29 isolates were HAdV 2. However, between December 2009 and February 2010, 5 (56%) of the 9 isolates identified during that time period were HAdV-2 (p <0.05). Thus, HAdV-2 was observed to be prevalent throughout the study period, whereas HAdV-3 was observed intensively from December 2008 to August 2009, and its frequency diminished with time.

### Phylogenetic analysis

**Figure 3:**
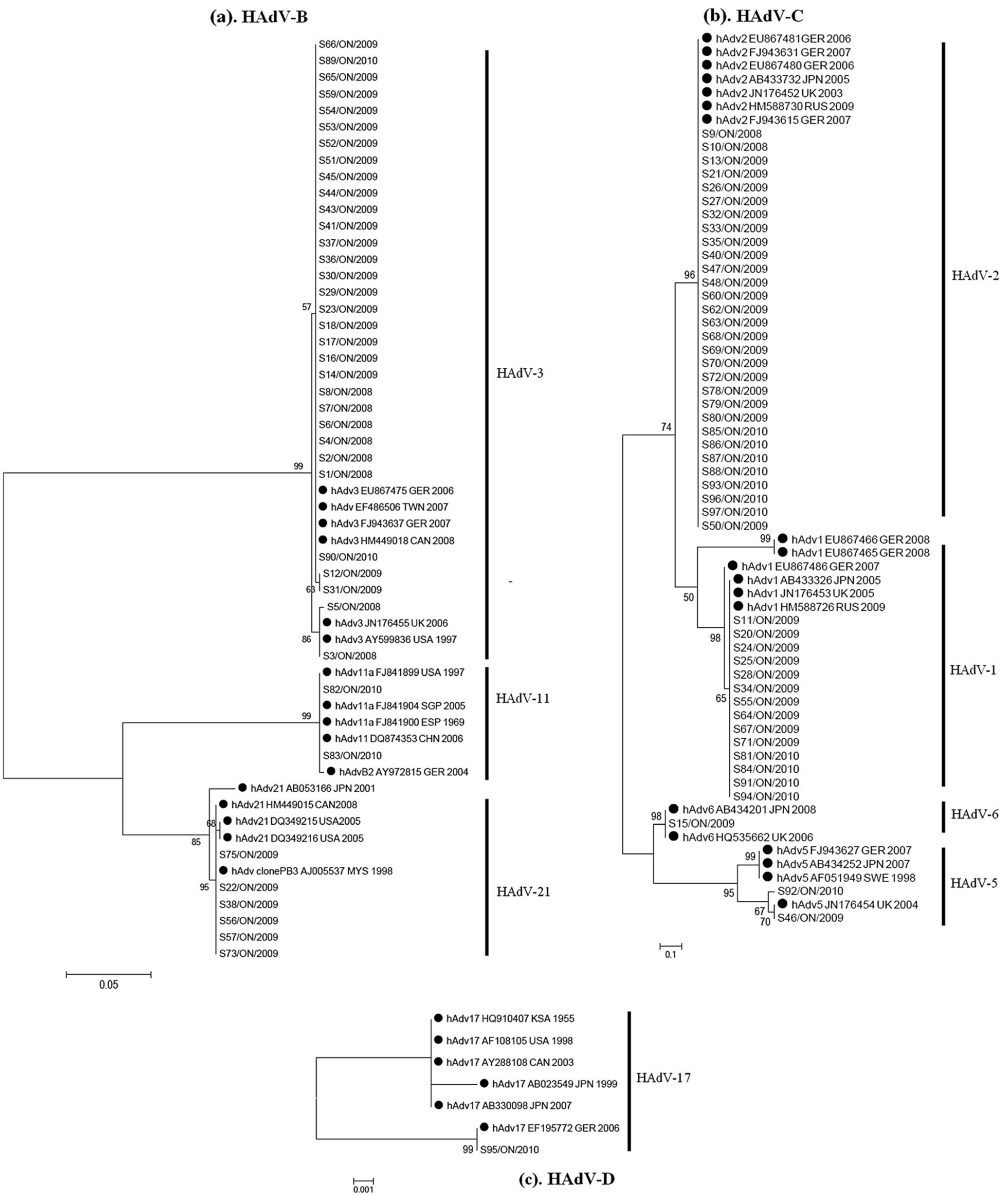
**Phylogenetic trees of Greater Toronto Area human adenovirus species B, C and D hexon gene, hypervariable region 7 (HVR 7), nucleotide sequences.** (**a**). HAdV B species and corresponding serotypes (3, 11, 21), (**b**). HAdV C species and corresponding serotypes (1, 2, 5, 6), and (**c**). HAdV D species and corresponding serotype (17). Reference sequences representing known serotypes are indicated by a solid circle. Multiple sequences alignments and phylogenetic trees were constructed using ClustalW and the maximum likelihood method algorithm within MEGA 5.05 software, respectively. Tree topology was supported by bootstrap analysis with 1000 pseudo replicate datasets. The scale bar represents the number of nucleotide substitutions per site between close relatives.

For the phylogenetic analysis, hexon gene sequences (ranging from 503 to 599 nucleotides) of the GTA HAdV isolates included in this study, along with reference sequences of HAdV species and serotypes were analyzed. Serotype identity was determined based on nucleotide similarity of ≥ 98% compared to respective reference sequences, all of which were included in the phylogenetic tree (Figure 3). Intraserotypic nucleotide sequence identities varied for different serotypes when comparing our study isolates to each other, with a range of 95–99%. Sequence identities for HAdV-1 (97–100%), HAdV-2 (95–99%), HAdV-3 (97–99%), and HAdV-21 (97–99%), varied. HAdV-5 and HAdV-11 showed identities of 97% and 99.3%, respectively among the 2 isolates belonging to each serotype. Intraserotypic sequence identities were not available for HAdV-6 and HAdV-17, as only one isolate belonged to each serotype.

## Discussion

We have observed a significant change in the circulating HAdV serotypes in the GTA of Ontario during December 2008 to April 2010. Of note is a significant decline in HAdV-3 and an increase in HAdV-2 in the population during the study period. These findings demonstrate that multiple serotypes would need to be included in order to create an effective HAdV vaccine. PHOL-Toronto is responsible for testing most, but not all cases of HAdV in the GTA; there are other tertiary institutions that test their own samples, and do not send them to PHOL. Thus, our results are not fully representative of all of the GTA’s HAdV cases. In addition, the generalizability of this study was further limited due to the fact that we were only able to sequence 90 (39%) of the 229 HAdV isolates that were detected at PHOL-Toronto during the study period, since only samples that were tested by viral culture were included. PHOL does not perform culture on samples from outbreaks or ICU patients, as these are tested by multiplex molecular methods. Thus, our sample was biased towards patients with less severe infections.

Yeung *et al.* showed that the prevalent serotypes in Ontario from September 2007 till June 2008 were HAdV-3 (44, 46%), HAdV-2 (25, 26%), HAdV-1 (17, 18%) and HAdV-21 (5, 5%) [3]. Our study demonstrates a similar serotype distribution with HAdV-3 (34, 38%), HAdV-2 (30, 33%), HAdV-1 (14, 16%) and HAdV-21 (6, 7%) also being most prevalent. We also documented a significant decline in HAdV-3 infections in the final 4 months of the study, suggesting a change in serotype distribution. Chen *et al.* also documented a marked shift in circulating serotypes in consecutive seasons in hospitalized children in southern Taiwan between 2001 and 2002 [9]. In 2001, HAdV-4 was found in the majority (57%) of isolates, while HAdV-3 was rare (5%) [9]. In 2002, while HAdV-3 became the major type (46%), the previously predominant HAdV-4 decreased to 6 per cent, and HAdV-7 increased from 2 to 19 per cent [9].

The cause for this seasonal change in serotype is yet unknown. One hypothesis could be due to unknown environmental factors, such as humidity and temperature. Another could be possible favourable or unfavourable interactions with other circulating respiratory viruses. It may also be that a build-up of immunity in the population could contribute to the decline in a serotype; in contrast, waning population level immunity to certain serotypes may result in an increase in prevalence/incidence.

As we observed, the previous study at this laboratory showed that the majority, 70 (73%) of the 96 isolates, were from children ≤ 4 years of age [3]. These findings are consistent with other studies that have also shown that pediatric populations with acute respiratory diseases are commonly infected with HAdV-1, HAdV-2, or HAdV-3 [10]. Furthermore, the literature also demonstrates that most adult populations with HAdV infections are predominantly infected with HAdV-3, HAdV-4, HAdV-7 and HAdV-11, with a few HAdV-1, HAdV-2, HAdV-6 and HAdV-14 cases [11]. Of note in this study, HAdV-11, HAdV-17 and HAdV-21 were only found among the adult population (age ≥ 18 years).

Adenovirus respiratory infections are highly prevalent in children; they have been documented in 5 to 11% of upper respiratory tract infections and bronchitis, 4 to 10% of pneumonia and pharyngitis, 2 to 10% of bronchiolitis, and 3 to 9% of croup in children [6]. Most outbreaks of adenovirus respiratory infections, especially in children < 7 years of age, have been caused by HAdV-3, HAdV-4, HAdV-7 and HAdV-21 [6]. Notably two of these serotypes, namely HAdV-3 and HAdV-21, have been circulating in the GTA for the past few years, as observed in this study and by Yeung *et al*[3].

From 1971 until 1996 the United States (US) had live oral adenovirus vaccines against HAdV-4 and HAdV-7 available for members of the military [12]. The vaccines were effective in preventing adenovirus infections; in fact, the overall ARD levels decreased by 50% to 60% among military recruits [12]. Studies conducted on the serum of vaccinated recruits have demonstrated a small cross-protective effect of the HAdV-4 and HAdV-7 vaccines against HAdV-3 and HAdV-14 [12,13]. Following the withdrawal of the adenovirus vaccination program, there was an outbreak of HAdV-14 in US military training camps in 2006 [12]. HAdV-14 had never been observed in a US recruit before 2006 [13,14]. These outbreaks have sparked recognition of the importance of continued adenovirus surveillance as well as a need for a vaccine [14]. In fact, due to a reoccurrence of HAdV outbreaks among military personnel, a second generation live oral vaccine against HAdV-4 and HAdV-7 was approved by the US Food and Drug Administration (FDA) in March 2011 [15]. As of October 2011, this vaccine is being administered to US military recruits [15].

Our findings suggest that for the development of a new vaccine, more serotypes would have to be included, since the prevalence of HAdV serotypes may change. The rapid shift in serotypes from season to season suggests that a more successful vaccine will be serotype independent, and directed against common epitopes shared by all serotypes. Continued molecular epidemiological surveillance of circulating adenovirus serotypes is a critical tool in monitoring the changes in HAdV serotypes causing clinical disease.

## Conclusions

There was a gradual shift in prevailing adenovirus serotypes during the 17 month study period, from predominantly HAdV 3 to HAdV 2. If an adenovirus vaccine were to be broadly implemented, multiple serotypes should be included.

## Competing interests

The authors declare that they have no competing interests

## Authors’ contributions

KZA performed the PCR, sequencing, and compiled the data. EL instructed and taught KZA, performed some sequencing, and bought all supplies. VRD performed the phylogenetic analysis. RO conducted data acquisition. RRH conceived the study. JBG supervised the study, and conducted its design and coordination. KZA, EL, VRD, RO, RRH, JBG drafted the manuscript and conducted data analysis. All authors have read and approved of the final manuscript.

## References

[B1] Pabbaraju K Wong S Fox J Detection of adenoviruses Methods MolBiol 2011 665 1 15 10.1007/978-1-60761-817-1_1 21116792

[B2] Sarantis H Johnson G Brown M Petric M Tellier R Comprehensive detection and serotyping of human adenoviruses by PCR and sequencing J Clin Microbiol 2004 42 9 3963 3969 10.1128/JCM.42.9.3963-3969.2004 15364976 PMC516336

[B3] Yeung R Eshaghi A Lombos E Blair J Mazzulli T Burton L Drews S Characterization of culture-positive adenovirus serotype from respiratory specimens in Toronto, Ontario, Canada: September 2007- June 2008 Virol J 2009 6 11 10.1186/1743-422X-6-11 19171030 PMC2656483

[B4] Ison M Adenovirus infections in transplant recipients Clin Infect Dis 2006 43 3 331 339 10.1086/505498 16804849

[B5] Houng H Gong H Kajon A Jones M Kuschner R Lyons A Lott L Lin K Metzgar D Genome sequences of human adenovirus 14 isolates from mild respiratory cases and a fatal pneumonia isolated during 2006–2007 epidemics in North America Respir Res 2010 11 11 116 20738863 10.1186/1465-9921-11-116 PMC2933684

[B6] Ghanaiem H Averbuch D Koplewitz B Yatsiv I Braun J Dehtyar N Wolf D Mandelboim M Engelhard D An outbreak of adenovirus type 7 in a residential facility for severely disabled children Pediatr Infect Dis J 2011 30 11 948 952 10.1097/INF.0b013e31822702fe 21694661

[B7] Tamura K Peterson D Peterson N Stecher G Nei M Kumar S MEGA5: molecular evolutionary genetics analysis using maximum likelihood, evolutionary distance, and maximum parsimony methods Mol Biol Evol 2011 28 10 2731 2739 10.1093/molbev/msr121 21546353 PMC3203626

[B8] Kimura M A simple method for estimating evolutionary rate of base substitutions through comparative studies of nucleotide sequences J Mol Evol 1980 16 111 120 10.1007/BF01731581 7463489

[B9] Chen H Chiou S Hsiao H Ke G Lin Y Lin K Jong Y Respiratory adenoviral infections in children: a study of hospitalized cases in southern Taiwan in 2001–2002 J Trop Pediatr 2002 50 279 284 15510759 10.1093/tropej/50.5.279

[B10] Hong JY Lee HJ Piedra PA Choi EH Park KH Koh YY Kim WS Lower respiratory tract infections due to adenovirus in hospitalized Korean children: epidemiology, clinical features, and prognosis Clin Infect Dis 2001 32 10 1423 1429 10.1086/320146 11317242

[B11] Guo L Gonzalez R Zhou H Wu C Vernet G Wang Z Wang J Detection of three human adenovirus species in adults with acute respiratory infection in China Eur J Clin Mircrobiol Infect Dis 2012 31 6 1051 1058 10.1007/s10096-011-1406-8 PMC7087767 21964587

[B12] Metzgar D Osuna M Kajon A Hawksworth A Irvine M Russell K Abrupt emergence of diverse species b adenoviruses at US military recruit training centers J Infect Dis 2007 196 1465 1473 10.1086/522970 18008225

[B13] Tate J Bunning M Lott L Lu X Su J Metzgar D Brosch L Panozzo C Marconi V Faix D Prill M Johnson B Erdman D Fonseca V Anderson L Widdowson M Outbreak of severe respiratory disease associated with emergent human adenovirus serotype 14 at a US air force training facility in 2007 J Infect Dis 2009 199 1419 1426 10.1086/598520 19351260

[B14] Binn L Sanchez J Gaydos J Emergence of adenovirus type 14 in US military recruits-a new challenge J Infect Dis 2007 196 1436 1437 10.1086/522969 18008220

[B15] Potter R Cantrell J Mallak C Gaydos J Adenovirus-associated deaths in US military during postvaccination period, 1999–2010 Emerg Infect Dis 2012 18 3 507 509 10.3201/eid1803.111238 22377242 PMC3309579

